# House dust mite allergen avoidance strategies for the treatment of allergic asthma: A hypothesis-generating meta-analysis

**DOI:** 10.1016/j.waojou.2024.100919

**Published:** 2024-06-11

**Authors:** Frank E. van Boven, Gert-Jan Braunstahl, Lidia R. Arends, Maurits S. van Maaren, Wichor M. Bramer, Roy Gerth van Wijk, Nicolette W. de Jong

**Affiliations:** a Department of Internal Medicine, Section of Allergology & Clinical Immunology, Erasmus MC, University Medical Center Rotterdam, P.O. Box 2040, 3000 CA, Rotterdam, the Netherlands; b Department of Pulmonology, Franciscus Gasthuis & Vlietland, P.O. Box 10900, 3004 BA, Rotterdam, the Netherlands; c Department of Pulmonary Medicine, Erasmus MC, University Medical Center Rotterdam, P.O. Box 2040, 3000 CA, Rotterdam, the Netherlands; d Department of Psychology, Education & Child Studies, Erasmus University Rotterdam, P.O. Box 1738, 3000 DR, Rotterdam, the Netherlands; e Department of Biostatistics & Epidemiology, Erasmus MC, University Medical Center Rotterdam, P.O. Box 2040, 3000 CA, Rotterdam, the Netherlands; f Medical Library, Erasmus MC, University Medical Center Rotterdam, P.O. Box 2040, 3000 CA, Rotterdam, the Netherlands

**Keywords:** Asthma, Environment, Hypersensitivity, Meta-analysis, Pyroglyphidae

## Abstract

**Background:**

This study continues the review by Gøtzsche and Johansen (Cochrane Database of Systematic Reviews, 2008, Art. No: CD001187), aiming to systematically generate hypotheses on the effectiveness of (sub)strategies for house dust mite allergen avoidance in the treatment of allergic asthma.

**Methods:**

We used the trials previously analysed by Gøtzsche and Johansen and searched recently published studies. Data on asthma symptom scores (ASS), ACQ, number of improved patients, AQLQ-scores, medication use, FEV_1_%, PC_20_, and FeNO levels were analysed. The effectiveness of strategies was assessed using Metafor in R.

**Results:**

Thirty-five trials involving 2419 patients were included in the final study. The patient-reported outcome number of patients with improved condition following total bedroom control was RR = 3.39 (95% confidence interval: 1.04 to 11.04, P = 0.04). The mean differences in the ASS by nocturnal air purification was −0.7 (95% confidence interval: −1.08 to −0.32, P < 0.001). Other outcomes including partial bedroom control were non-significant or clinically not of importance.

**Conclusions:**

Total bedroom control and nocturnal air purification of the breathing zone hypothetically provides clinical benefits in patients with house dust mite-induced allergic asthma. The number of patients with improvements in their condition respectively the asthma symptom score differences showed potential in small subgroups, consisting of single studies. Partial bedroom control is not recommended.

**Systematic Review Registration:**

Prospero CRD42022323660.

## Introduction

Asthma is a major public health problem, affecting more than 250 million people worldwide.^1^ Asthma is characterised by reversible airflow obstruction associated with airway hyperresponsiveness and increased secretion of mucus.^2^ Various environmental factors interact with the airways, causing acute and chronic inflammation.^2^ Approximately 50–110 million patients are sensitized to house dust mite allergens which can trigger allergic asthma.^3^ House dust mite allergen-related immune reactions suggest that allergen avoidance is the cornerstone of allergic asthma treatment. However, the therapy of house dust mite allergen avoidance became controversial after a Cochrane review by Gøtzsche and Johansen was published.^4^ They concluded “methods aimed at reducing exposure to house dust mite allergen cannot be recommended”. The dominating meta-analysis included 56 randomised controlled trials to investigate considerably varying environmental interventions.^5^ In total, 3121 patients with predominantly mild to moderate allergic asthma were studied. In approximately 50% of the trials, co-sensitisation to other allergens was reported.^6^ Over the years, the conclusions from this Cochrane review have been adopted by multiple international and national guidelines.^7^^,^^8^ Recently, an editorial expressed concern regarding the conclusions from the Cochrane-review by Gøtzsche and Johansen,^4^ stating the following: “This 2011 review predates current reporting standards and methodological expectations for Cochrane Reviews. It should not be used for clinical decision-making” (https://doi.org/10.1002/14651858.CD001187.pub3, assessed September 15th, 2023). Nevertheless, this editorial note does not affect the need for an evidence-based study addressing whether reducing exposure to house dust mite allergens might benefit patients with asthma.^9^

Previously defined strategies for house dust mite allergen avoidance have recently been reintroduced.^5^ Total avoidance^10^ and high-altitude and climate treatments^11^ were initially described, and are both well-accepted for their clinical benefits. Textile-related strategies include exposure-based control and concurrent bedroom interventions. Exposure-based control is when the choice of interventions is based on the assessment of actual indoor exposure, as defined by Bronswijk.^12^ Whereas, concurrent bedroom interventions are a set of a priori defined interventions which primarily aim to treat the sleeping environment, as defined by Colloff.^13^ Regarding concurrent bedroom interventions, comprehensive sets of interventions have resulted in an ascending reduction in mite allergen load from a mattress.^14^ However, this hypothesis remains to be confirmed. Notably, the psychrometric control of house dust mites is not considered a textile-related strategy as it aims to lower mite numbers but not the allergen load.^5^ An alternative strategy directly related to the breathing zone is air purification,^15^ including the delivery of temperature-controlled laminar airflow during sleep.^16^

A key component of a review question is to specify the interventions. When specifying interventions, an initial question is whether the treatments have variation.^17^ The effects of variations in the intervention are analysed by subgrouping or regression. However, the pitfall of subgroup analyses and meta-regression is drawing false positive conclusions due to loss of power.^18^ It is recommended that the primary aim in investigating whether varying the intervention affects the health outcomes is to define the protocol.^18^ This study continues the meta-analysis by Gøtzsche and Johansen,^4^ with the aim to generate hypotheses on the effects of (sub)strategies for house dust mite avoidance in the treatment of allergic asthma. Our choices of outcomes follow that of Gøtzsche and Johansen.^4^

## Methods

### Reference searches

The first group of trials included in this study were obtained from the systematic review by Gøtzsche and Johansen^4^ and was labelled as reference group A. The group comprised 56 trials. An updated search group (group B) comprised trails collated from Embase.com, MEDLINE via Ovid, and Cochrane Central via Wiley and the trial search was performed by an experienced medical information specialist (WB). Trail collection ceased on January 12, 2024. The search consisted of terms for house dust mites or house dust combined with terms for air quality or environmental exposure Search terms in Embase:

('Pyroglyphidae'/exp OR 'mite'/de OR 'Acari'/de OR 'house dust'/de OR 'house dust allergen'/de OR 'mite infestation'/de OR 'house dust allergy'/de OR 'dust exposure'/de OR (Dermatophagoid∗ OR mite OR mites OR 'D farinae' OR 'd pteronyssinus' OR Pyroglyphid∗ OR Euroglyph∗ OR 'e maynei' OR Acari∗ OR housedust∗ OR (dust NEAR/6 (allerg∗ OR sensiti∗ OR hypersensiti∗ OR indoor∗ OR house∗ OR domestic∗ OR asthma∗ OR ambient∗))):ab,ti) AND ('air conditioning'/de OR 'exposure'/de OR 'dust exposure'/de OR 'environmental exposure'/de OR 'environmental parameters'/de OR 'avoidance behavior'/de OR 'environmental factor'/de OR 'environmental management'/de OR 'textile'/de OR 'home environment'/de OR 'tertiary prevention'/de OR 'microclimate'/de OR 'room ventilation'/de OR 'air quality'/de OR 'ambient air'/de OR 'air quality control'/de OR humidity/de OR 'environmental sanitation'/de OR 'sanitation'/de OR (avoidance∗ OR (impermeab∗ NEAR/3 cover∗) OR ((humid∗ OR allergen∗ OR climate∗) NEAR/3 (control∗ OR reduction∗)) OR (air NEAR/3 (condition∗ OR filt∗ OR qualit∗ OR ambient∗ OR control∗ OR clean∗)) OR ventilat∗ OR expos∗ OR textile∗ OR load OR environment∗ OR (dust NEAR/3 level∗) OR anti-mite OR spray∗ OR mattress∗ OR management∗ OR (tertiary NEAR/3 prevent∗) OR microclimate∗ OR micro-climate∗ OR sanitation OR bed-cloth∗ OR bed-cover∗ OR bedding OR furnish∗):ab,ti) AND ('Controlled clinical trial'/exp OR 'Crossover procedure'/de OR 'Double-blind procedure'/de OR 'Single-blind procedure'/de OR (random∗ OR factorial∗ OR crossover∗ OR (cross NEXT/1 over∗) OR placebo∗ OR ((doubl∗ OR singl∗) NEXT/1 blind∗) OR assign∗ OR allocat∗ OR volunteer∗ OR trial OR groups):ab,ti) NOT ([animals]/lim NOT [humans]/lim) NOT ([Conference Abstract]/lim) AND [English]/lim), and was filtered to only include clinical trials using the Cochrane search filter. Studies focused on asthmatic patients not primary sensitized to house dust mite, examples given cat or dog, were not included by the search. Conference abstracts and articles published in languages other than English were excluded. The first author (FB) and last author (NJ) screened the titles and/or abstracts to identify randomised trials that met the inclusion criteria, using the method described by Bramer et al.^19^ The full texts of these potentially eligible trials were retrieved and assessed for inclusion in reference group B by FB and NJ. Any ambiguities in selection were resolved through a discussion between FB and NJ.

### Selection of studies

Trials from both reference group A and the updated search (group B) were (re)selected for inclusion using the following criteria, as described by Boven et al.^6^

− This was a randomised, placebo-controlled trial with anonymizing. Although some mite control interventions are not possible or are very difficult to anonymize, we accepted trials that included washing instructions for bedsheets or removal of soft toys.
− The study was a peer-reviewed publication with full text present (not a conference abstract).
− This manuscript was published in English.
− All participants studied were diagnosed with house dust mite-induced allergic asthma by a physician. This includes participants whose sensitisation was assessed by either skin testing or serum assays for specific IgE antibodies (house dust mite allergies). Asthma assessment included a review of the patient's history of asthma symptoms and pulmonary function tests.
− This intervention was designed to reduce exposure to mite antigens at home for the treatment of asthma (monotrigger therapy). This could include 1 of the following sub-strategies:^5^^,^^14^
  • Partial bedroom control (maximum of 2 interventions: fitting of mite-impermeable covers to all bedding elements and/or laundering of bedding monthly with hot water, with a minimum temperature of 60 °C).
  • Total bedroom control (at least 3 interventions: partial bedroom control concurrent with the removal or cleaning of the bedroom carpet, soft toys, and other textiles).
  • Air purification using portable units randomly placed in a bedroom, aiming to purify the indoor air of the entire room.
  • Nocturnal air purification of the breathing zone.
  • Interventions that were not classified as a strategy (non-classified interventions that did not fit the previous sub-strategies).

The recommended 90% reduction of the mite load^20^ was achieved by a combination of three-bedroom interventions (total bedroom control).^14^ Therefore, we grouped the concurrent bedroom interventions into two sub-strategies (partial and total bedroom control). The air purification strategies group included the use of portable air purifiers and nocturnal air purification. Nocturnal air purification reduces the number of airborne particles emitted into the breathing zone with likely a 100-fold more than those of a room air cleaner.^21^

A flow chart depicting the updated search strategy and inclusion criteria of studies was created using the Preferred Reporting Items for Systematic Reviews and Meta-Analyses diagram.^22^

### Preventing bias in outcomes

Data extraction is subject to several potential biases and imprecise outcome summary (OS) statistics. One issue is the choice of final values or change scores for meta-analysis of continuous asthma outcomes using summary statistics. The final or changed values were extracted according to the recommendations published by Egbewale et al.^23^ The final values were (re)extracted for the ASS, ACQ, AQLQ, and PC_20_, and numbers improved. Concurrently, the change scores for FEV_1_, medication use, and FeNO levels were (re)extracted. Two other aspects threatening the extraction of unbiased summary statistics are the mixing of patient- and physician-reported judgments and the use of interim data. Outcomes on subjective judgments, such as well-being, are useful for describing discrete observations; however, they promote a potential risk of detection bias when mixing patient-reported and physician-reported observations.^24^ Another well-studied type of bias in reviews on avoidance occurs with the use of interim data (selective reporting). This data may result in a biased estimation of the treatment effect.^25^^,^^26^ We reviewed the data extracted to assess whether subjective or objective measures (asthma symptom score and numbers improved) or the use of interim data were included. If necessary, data were re-extracted to exclude any possible bias.

When trials included multiple intervention arms, the intervention ranking highest in the strategy of concurrent bedroom interventions was selected for extraction (Supplement A, Table S3), as implemented by Higgins et al.^17^ The outcome regarding numbers improved was extracted by continuing the method used by Gøtzsche and Johansen,^4^ which involved summarising categorical data of the subjective outcome of well-being (defined as the number of patients who reported their condition had improved). Also, the numbers improved can reflect on the categorical result of the ASS.^4^ These outcomes were sub-grouped into patient-reported (PRO) and physician-reported numbers. The number was considered not to improve for mathematical reasons when a trial reported a decrease in the number of patients. The improved zero number was set to 0.5.

Although the exposure at baseline varied considerably in the previous trials,^6^ load is primarily a suboptimal proxy for airborne exposure.^27^^,^^28^ Both high- and low-load levels can result in high airborne exposure.^29^ Therefore, in this study we did not account for the mite allergen load from the mattress (μg/g of dust).

### Data collection and analysis

#### Data collection

FB and NJ elaborated on the control and extraction of data. Ambiguities were resolved through discussion between the two researchers. The outcomes included both main and additional outcomes.

##### Main outcomes

The main outcomes assessed were as follows:

− Asthma symptom score (ASS).
− Asthma control questionnaire (ACQ) scores for adults and children.^30^^,^^31^
− The number of patients improved (numbers improved).
− The asthma quality of life questionnaire (AQLQ)^32^ and paediatric asthma quality of life questionnaire (PAQLQ).^33^
− The medication usage.
− Percentage of predicted FEV_1_ (forced expiratory volume in 1 s) (%).
− Log-transformed PC_20_ (histamine or methacholine concentration that caused a 20% decrease in FEV_1_).
− Fractionated exhaled NO levels (FeNO).

Minimal clinically important differences (MCID) have been defined in multiple asthma outcomes, sometimes at varying magnitudes. These include an improvement of 0.5 points in the ACQ,^34^ an improvement of 0.5 points in the AQLQ,^35^ an increase in FEV_1_ from 10% to 20%,^34^^,^^36^ and a decrease of 20% in FeNO (approximately corresponding to a raw change up to 8 ppb).^37^

##### Additional outcomes

The additional outcomes assessed were as follows:

− Sub-strategies for house dust mite avoidance
− Patient- and physician-reported measures
− Use of interim data (yes or no)
− Patient type (child or adult)
− Presence of multiple sensitisers (yes or no)

### Data analysis

The effect size was set to the standardised mean difference (SMD), except for the numbers that improved (risk ratio [RR]). When the SMD was significant, we also assessed the mean difference (MD) for clinical interpretation, when possible. First, the effect size of the health outcomes, overall and differentiated by the (sub)strategies, was estimated by a random-effects meta-analysis including the Knapp and Hartung adjustment.^39^ The Knapp and Hartung adjustment was not included when observing no between-study variance (I^2^ = 0%) in the presence of ≤5 trials.^40^ The effect size for RR was calculated using the Mantel–Haezel approach. Additionally, I^2^ was calculated to examine heterogeneity in the outcomes. The results were visualised using forest plots. All calculations and forest plots were performed using the Metafor package,^41^ version 4.2.0 in R, version 4.2.3.^42^ We performed an omnibus test without an intercept to test for differences between subgroups. We also calculated the studentized residuals for every outcome (r_s_, an influential diagnostic indicating whether an observation was significantly removed from the centre of the data). The explanatory variables of interest included possible confounding by the type of patient (child/adult) and the presence of co-sensitisation. These explanatory variables were analysed for a minimum of ten trials per variable.^17^ The level of significance was set at α = 0.05.

### Risk of bias assessment

The risk of bias was assessed for the following domains: random sequence generation, allocation concealment, blinding quality, incomplete outcome data, and selective outcome reporting. The assessments were performed by FB and NJ, respectively, and presented using the Robvis tool.^38^ Any ambiguities in the assessment were resolved through a discussion between the 2 researchers.

## Results

### Selection of studies

**Fig. 1:**
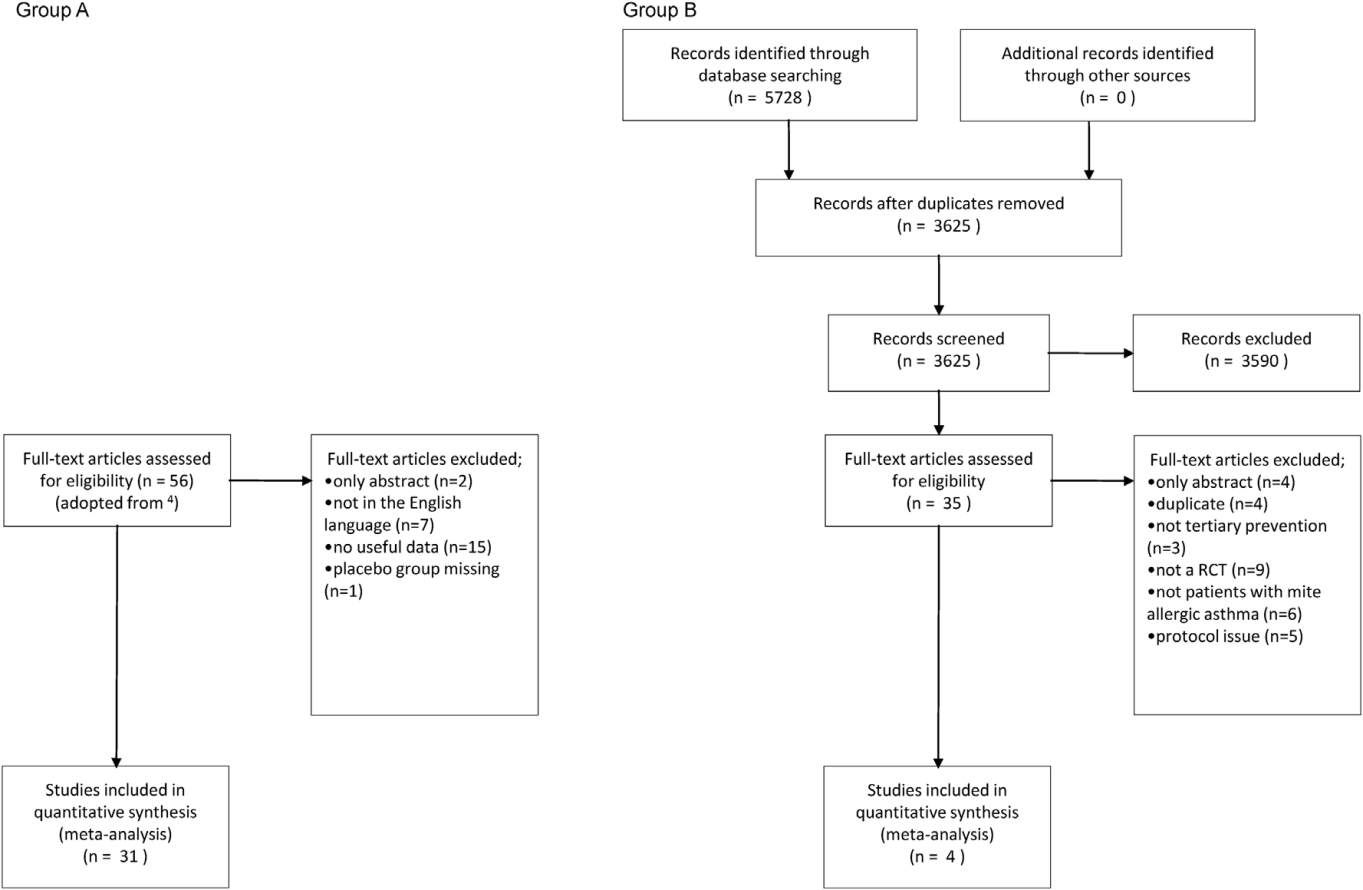
Flow chart of the updating literature search and selection of studies (reference group A respectively B). RCT, randomised controlled trial

The selection and inclusion of the studies resulted in two groups. In group A, we included 31 trials from the existing meta-analysis by Gøtzsche and Johansen (reference search till July 2011, Fig. 1A).^4^ Group B included results from our updated reference search (Fig. 1B). We found 3625 titles and abstracts published till January 12th, 2024. Three thousand five-hundred ninety were excluded for not reporting a randomised controlled trial on the treatment of asthma by house dust mite allergen avoidance. Thirty-five potentially relevant titles were selected for inclusion. Thirty-one references in Group B were excluded because they did not meet our inclusion criteria (Supplement A, Table S1, S2). Totally, 35 full**-**text publications were included in our analysis.^43^, ^44^, ^45^, ^46^, ^47^, ^48^, ^49^, ^50^, ^51^, ^52^, ^53^, ^54^, ^55^, ^56^, ^57^, ^58^, ^59^, ^60^, ^61^, ^62^, ^63^, ^64^, ^65^, ^66^, ^67^, ^68^, ^69^, ^70^, ^71^, ^72^, ^73^, ^74^, ^75^, ^76^, ^77^

### Description of the included trials and their baseline characteristics

**Fig. 2:**
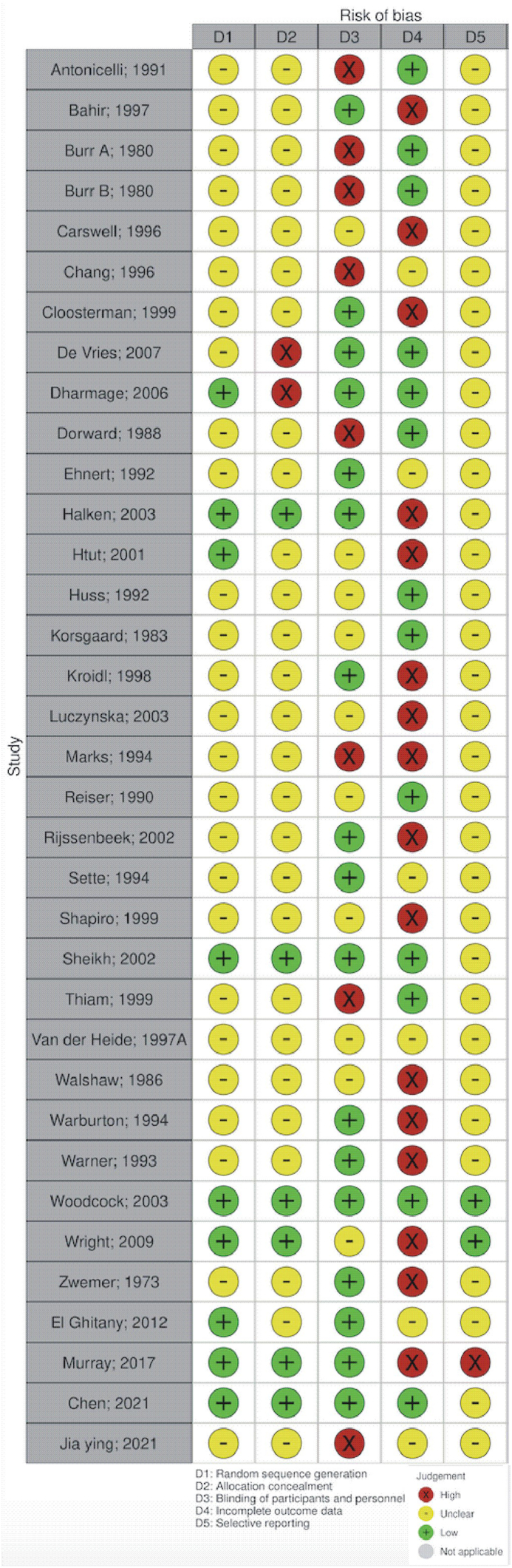
Summary of the risk of bias judgements across the 35 randomised controlled trials

Thirty-five trials published between 1973 and 2021 reported the treatment of house dust mite-induced allergic asthma by avoidance, including 2419 patients (Table 1). Of the 35 included trials, 12 reported on the strategy of concurrent bedroom interventions, including two-by-total bedroom control.^47^^,^^62^ Four trials reported air purification strategies, including one using nocturnal air purification.^73^ Nineteen trials studied an unclassified intervention with varying treatments (mostly by providing the patients an acaricide (spray or powder),^44^ but also a set of concurrent bedroom interventions was reported,^74^ not meeting the definition by Colloff^13^). Fifteen trials reported on the treatment of children, and 20 on the treatment of adults. In 16 trials, co-sensitisation at baseline to pollen, cat, and/or dog was described, 5 trials reported mono-sensitisation, and 14 trials did not report this information (NA). Asthma symptom scores were reported in 14 trials. The standardised mean score (mean divided by maximum number of the score) at baseline was 0.12 (95% CI: 0.08 to 0.16; n = 380; trials: 8; I^2^ = 99.9%). Four trials reported on the ACQ (mean at baseline, 1.05 (95% CI: 0.49 to 1.60; n = 416; trials: 4; I^2^ = 96%). Only one trial reported on the AQLQ by use of the PAQLQ questionnaire (mean at baseline 5.46; 95% CI: 5.22 to 5.70; n = 120). The percentage of predicted FEV_1_ was reported in 14 studies. The mean value at baseline was 85.1% (95% CI: 80.2–89.9%; n = 759; trials: 14; I^2^ = 97%). Thirteen trials published measurements on PC_20_, with a mean at baseline of 1.44 mg/mL (95% CI: 0.30–2.58 mg/mL; n = 417; trials: 10; I^2^ = 99%). Finally, the FeNO was reported in one trial (mean value at baseline 56 ppb; 95% CI: 45.3–66.7 ppb; n = 38). In the trial by Murray et al,^75^ they reported that the “GINA step had been increased in 10.7% of the active group and in 14.5% of the placebo group”. We processed this as no improvement was observed in either group. The risk of bias in 35 trials was predominantly judged to be unclear, particularly in the domains of random sequence generation and allocation concealment (Fig. 2, Supplement A Table S4).

**Table 1:** Study characteristics of 35 randomised controlled trials included in the meta-analysis.

Author; year	Sub-strategy assessed	Subjects	Size	Multiple allergies	Health outcomes extracted
Antonicelli; 1991^43^	Air purification - mobile	Adult	18	NA	FEV_1_, PC_20_
Bahir; 1997^44^	Not classified into a strategy	Child	30	NA	Asthma symptom score, numbers improved, FEV_1_
Burr; 1980A^45^	Not classified into a strategy	Child	53	NA	Numbers improved
Burr; 1980B^46^	Not classified into a strategy	Child	42	NA	Numbers improved
Carswell; 1996^47^	Total bedroom control	Child	49	Yes	Numbers improved, FEV_1_
Chang; 1999^48^	Not classified into a strategy	Adult	26	Yes	Asthma symptom score, FEV_1_, PC_20_
Cloosterman; 1999^49^	Partial bedroom control	Adult	157	Yes	Asthma symptom score, FEV_1_, PC_20_
De Vries; 2007^50^	Partial bedroom control	Adult	105	Yes	Asthma control questionnaire
Dharmage; 2006^51^	Partial bedroom control	Adult	30	Yes	Asthma symptom score, medication usage, PC_20_
Dorward; 1988^52^	Not classified into a strategy	Adult	18	Yes	FEV_1_, PC_20_
Ehnert; 1992^53^	Partial bedroom control	Child	16	NA	PC_20_
Halken; 2003^54^	Not classified into a strategy	Child	47	No	Medication usage, FEV_1_, PC_20_
Htut; 2001^55^	Not classified into a strategy	Adult	23	Yes	PC_20_
Huss; 1992^56^	Not classified into a strategy	Adult	52	No	Asthma symptom score, medication usage
Korsgaard; 1983^57^	Not classified into a strategy	Adult	46	Yes	Asthma symptom score, medication usage
Kroidl; 1998^58^	Not classified into a strategy	Adult	78	No	Numbers improved
Luczynska; 2003^59^	Partial bedroom control	Adult	31	Yes	Asthma symptom score
Marks; 1994^60^	Partial bedroom control	Adult	35	NA	Asthma symptom score, FEV_1_, PC_20_
Reiser; 1990^61^	Not classified into a strategy	Child	46	NA	PC_20_
Rijssenbeek; 2002^62^	Total bedroom control	Adult	30	No	Asthma symptom score, PC_20_
Sette; 1994^63^	Not classified into a strategy	Adult	24	Yes	PC_20_
Shapiro; 1999^64^	Not classified into a strategy	Adult	36	Yes	FEV_1_
Sheikh, 2002^65^	Partial bedroom control	Child	43	No	Asthma symptom score
Thiam; 1999^66^	Partial bedroom control	Child	12	NA	Asthma symptom score, FEV_1_
Van der Heide, 1997^67^	Not classified into a strategy	Adult	38	Yes	PC_20_
Walshaw; 1986^68^	Not classified into a strategy	Adult	42	NA	Medication usage, FEV_1_
Warburton; 1994^69^	Air purification - mobile	Adult	24	Yes	FEV_1_
Warner; 1993^70^	Not classified into a strategy	Child	28	NA	Asthma symptom score, medication usage
Woodcock; 2003^71^	Partial bedroom control	Adult	641	Yes	Asthma symptom score, medication usage
Wright; 2009^72^	Not classified into a strategy	Adult	100	Yes	Asthma control questionnaire, FEV_1_
Zwemer; 1973^73^	Air purification - nocturnal	Child	24	NA	Asthma symptom score
El-Ghitany; 2012^74^	Not classified into a strategy	Child	80	NA	Numbers improved, FEV_1_
Murray; 2017^75^	Partial bedroom control	Child	225	Yes	Asthma control questionnaire, asthma quality of life questionnaire, numbers improved
Chen; 2021^76^	Not classified into a strategy	Child	132	NA	Asthma control questionnaire
Jia ying; 2021^77^	Air purification - nocturnal	Child	38	NA	FeNO

FEV_1_, forced expiratory volume in 1 s; NA, not applicable

### Standardised effects sizes of the (sub) strategies

**Fig. 3:**
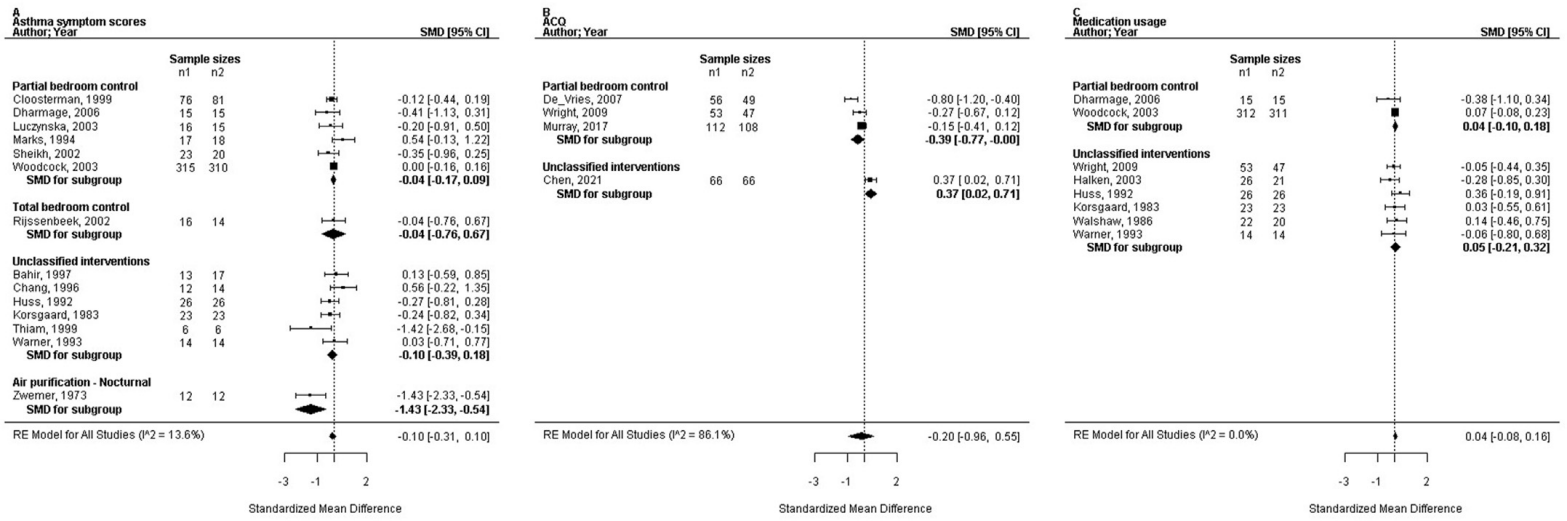
Forest plots of the standardised mean difference in clinical outcomes related to the different sub-strategies of concurrent bedroom interventions and air purification; (a) the asthma symptom scores, (b) the asthma control questionnaire, (c) the medication usage. ACQ, asthma control questionnaire; CI, confidence interval; SMD, standardised mean difference; RE, random-effects model

**Fig. 4:**
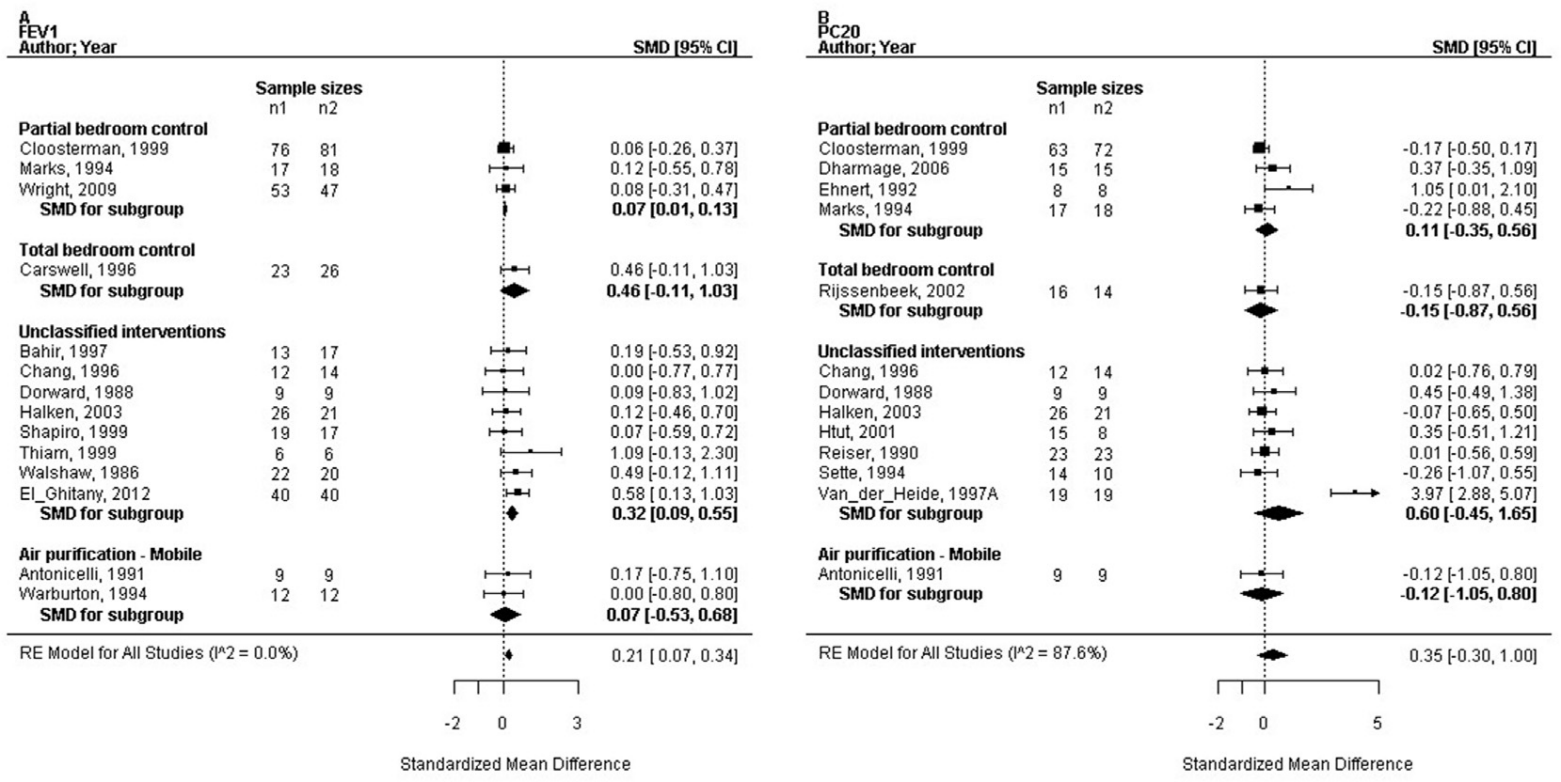
Forest plots of the standardised mean difference in physiological outcomes related to the different sub-strategies of concurrent bedroom interventions and air purification; (a) the forced expiratory volume in 1-s (FEV_1_), (b) the PC_20_. FEV_1_, forced expiratory volume in 1 s; CI, confidence interval; SMD, standardised mean difference; RE, random-effects model

**Fig. 5:**
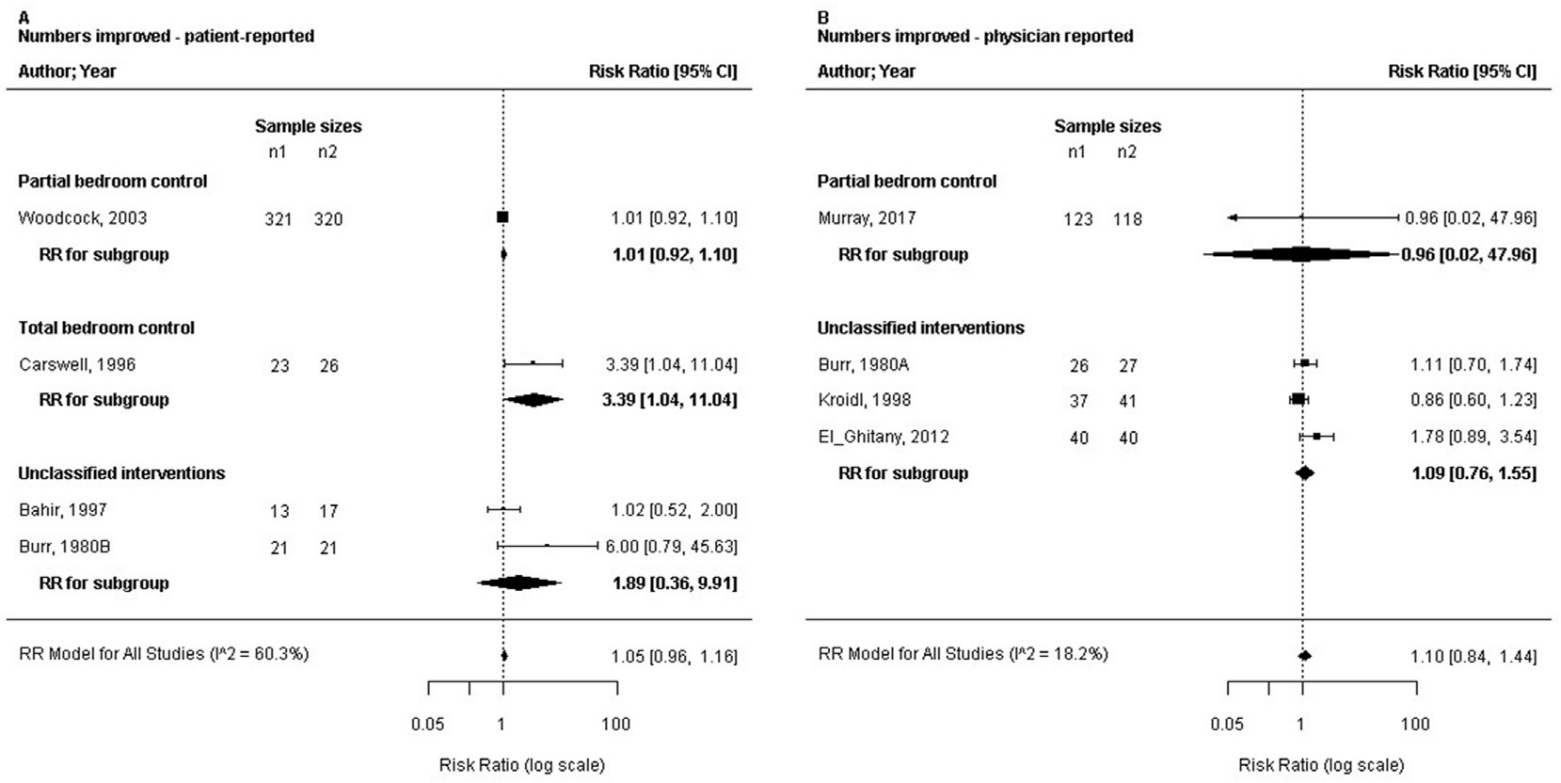
Forest plots of the risk ratio for the number of patients improved in relation to the different sub-strategies of concurrent bedroom interventions and air purification; (a) the patient-reported number of patients improved, (b) the physician-reported number of patients improved. CI, confidence interval; RR, risk ratio

The SMD in the FEV_1_ by the combined strategies of concurrent bedroom interventions and the air purification strategy was assessed increasing with +0.21 (95% CI: 0.07 to 0.34; n = 674; trials: 14; P = 0.006; I^2^ = 0%). Other standardised effects of the combined strategies were not significant (ASS, ACQ, AQLQ, PC_20_, medication use, and number of patients improved; Fig. 3, Fig. 4, Fig. 5). Ten trials reported on the partial bedroom control sub-strategy. The SMDs in ASS, ACQ, AQLQ, FEV_1_, PC_20_, medication usage and the outcome numbers improved were all not significant (P = 0.31 to 1.0), the I^2^ ranged from 0% to 86%. We assessed the RR in the PRO numbers improved by the sub-strategy of total bedroom control was 3.39 (95% CI: 1.04 to 11.04; n = 49; trials: 1; P = 0.04). The SMDs for other outcomes by this sub-strategy were not significant (P = 0.67, PC_20_, and P = 0.91 in ASS). In the subgroup of not classified interventions (19 trials), we assessed the SMD significantly increasing the FEV_1_ as +0.32 (95% CI: 0.08 to 0.56; n = 379; trials: 8; P = 0.02; I^2^ = 0%). The standardised ACQ was significantly improved with +0.37 (95% CI: 0.02 to 0.71; n = 132; trials: 1; P = 0.04). The effect sizes of the other outcomes were not significant (P = 0.17 to 0.89 for the ASS, AQLQ, PC_20_, medication usage, and numbers improved). Mobile air purification (two trials) did not show a significant effect size in FEV_1_ or PC_20_ (P = 0.55 respectively 0.80). Nocturnal air purification of the breathing zone (2 trials) resulted in a significant SMD in ASS (−1.43 [95% CI: −2.33 to −0.54; n = 24; trials: 1; P = 0.002]). Furthermore, the SMD for FeNO was not significant (P = 0.80).

### Additional analysis

For 3 statistically significant effect sizes, the data allowed for the assessment of an MD. The asthma symptom score results following nocturnal air purification of the breathing zone showed a decrease in MD of −0.7 at 4 weeks of treatment (95% CI: −1.08 to −0.32; n = 24; trials: 1; P < 0.001). In the ACQ, the MD provided by unclassified interventions had an increase of +0.2 (95% CI: 0.01 to 0.38; n = 132; trials: 1; P = 0.03). Data from the non-classified interventions did not allow for the assessment of MD in FEV_1_. The most influential trial in this subgroup^74^ reported an increase of +2.3% percentage predicted FEV_1_ (95% CI: 0.6 to 4.1; n = 80; trials: 1; P = 0.009). The number of trials available did not allow for subgrouping according to the type of patient (child/adult) and the presence of co-sensitisation (yes/no).

Another analysis yielded differences between the sub-strategies per outcome. In all of the outcomes except the FEV_1_ the omnibus test resulted in non-significant P-values (ASS: P = 0.18; ACQ: P = 0.29; FEV_1_: P = 0.02; PC_20_: P = 0.78; medication usage: P = 0.74; PRO numbers improved: P = 0.64; physician-reported numbers improved: P = 0.90). Studentized residuals were increased in four outcomes (ASS: r_s_ = −2.80 for the study by Zwemer et al;^73^ FEV_1_: r_s_ = 2.59 for the study by El-Ghitany et al;^74^ PC_20_: r_s_ = 8.2 for the study by Van der Heide et al;^67^ PRO numbers improved: r_s_ = 2.0 for the study by Carswell et al;^47^ and physician-reported numbers improved: r_s_ < 2.0 for all studies).

## Discussion

Randomised controlled trials with a focus on house dust mite allergen avoidance for the treatment of allergic asthma were grouped into the sub-strategies of concurrent bedroom interventions and air purification. Following this, we observed that total bedroom control resulted in three times more likely increase in the patient-reported number of patients with improved conditions compared with the placebo group (RR = 3.39; P = 0.04). Furthermore, nocturnal air purification of the breathing zone decreased the asthma symptom score substantially (MD = −0.7; P < 0.001). The standardised effect of FEV_1_ when implementing the combined strategies was small; however, the effect significantly increased (SMD = +0.21; P = 0.006). We included a heterogeneous subgroup of non-classified interventions to explain this increase (SMD = +0.32; P = 0.02). The data in this subgroup of unclassified interventions did not allow for assessing the MD in FEV_1_; however, we observed that the trials included which described the percentage predicted FEV_1_ reported an effect size smaller than the MCID. Consequently, we considered the significant observations in the FEV_1_ as clinically insignificant.^4^ Other effect sizes were non-significant or did not clinically meet the MCID, accordingly the findings by Gøtzsche and Johansen.

Heterogeneity was absent in the air purification strategies and considerable in concurrent bedroom interventions, showing great variation. This may have occurred because of multiple reasons. First, the small number of trials in the first subgroup may have played an important role. When assessing the differences between the subgroups, the omnibus tests for differences between the sub-strategies were not significant except for FEV_1_. The predominantly absence of significance in the omnibus tests could indicate that neither of the benefits observed would influence the average effect size when combining all sub-strategies. However, due to the loss of power in the subgroup analysis, the absence of significance in testing for differences between subgroups should not be interpreted as the true means in the subgroups being equal. Second, we observed that all studies strongly influencing an effect size (increased studentized residues r_s_ ≥ 2.0),^47^^,^^73^^,^^74^ were related to significant standardised effect sizes. Trials conducted by Carswell et al, El-Ghitany et al, and Zwemer et al^47^^,^^73^^,^^74^ were all environmentally characterised by extensive or precise interventions aimed at reducing airborne allergen exposure within the bedroom. This indicates that the largely different environmental characteristics between the sub-strategies could explain the observed benefits. Finally, the baseline characteristics we observed were in line with our previous observations,^6^ suggesting a majority of the patients had mild-to-moderate asthma.

As the debate on house dust mite avoidance is still dominated by the meta-analysis by Gøtzsche and Johansen,^4^ we specifically compared the contrasting results. Gøtzsche and Johansen^4^ reported that there was no benefit to any of their studied outcomes. Therefore, the ASS in the nocturnal air purification subgroup, FEV_1_ in other categories (undefined strategies), and the PRO numbers improved by total bedroom control are of great interest. Our study differed compared with the meta-analysis by Gøtzsche and Johansen^4^ in that we systematically and consequently (re)extracted unbiased data regarding the sub-strategies. The benefits of total bedroom control were fully explained in the trial by Carswell et al,^47^ who reported follow-up measurements at 2, 6, and 24 weeks. Comparatively, Gøtzsche and Johansen^4^ extracted interim-data at 6 weeks. This was because the reduction in house dust mite load was highest at 6 weeks. However, house dust mite load is a poor proxy for airborne allergen exposure.^28^ Therefore, in contrast, we extracted the data at the endpoint of the study and considered this measurement at 6 weeks as the interim value. The effect size of FEV_1_ within the unclassified interventions group was highly influenced by the trial by El-Ghitany et al.^74^ This trial observed a significant statistical change of +2.3% in the percentage predicted FEV_1_ (P = 0.009). Furthermore, they studied a combination of comprehensive methods which did not fit exactly within the sub-strategy of total bedroom control. Regarding the asthma symptom score, we observed benefits regarding nocturnal air purification described within the trial by Zwemer et al.^73^ In the meta-analysis by Gøtzsche and Johansen,^4^ this trial also showed benefits relating to nocturnal air purification (SMD = −1.43, 95% CI: −2.35 to −0.52, weight 1.3%). As we did subgroup environmentally different avoidance types, we could report this observation. In addition, regarding the outcome of PC_20_ we observed a very influential observation (provided within a trial by Van der Heide et al,^67^ r_s_ = 8.2). Although the sub-strategies in this outcome did not show significant results, this observation differed considerably in comparison to the study by Gøtzsche and Johansen,^4^ highlighting the crucial role of data extraction.

The hypothesis of interest developed in this study related to the sub-strategies of total bedroom control and nocturnal air purification in the breathing zone. Environmentally, the sub-strategy of total bedroom control combining the three interventions is superior to the other sub-strategies of concurrent bedroom interventions.^14^ The benefits of nocturnal air purification in the breathing zones of patients with allergic asthma have been shown in systematic reviews by Boven et al^78^ and Chauhan et al.^79^ Many recommendations have been made to restudy the clinical interventions for allergic asthma through house dust mite avoidance. Gøtzsche and Johansen^4^ highlighted a need for trials based on rigorous methods and with a low risk of bias. Examples of such trials include those by Sheikh et al,^65^ Woodcock et al,^71^ and Chen et al.^76^ Boven et al,^6^ recommended that increased focus is placed on patients with severe asthma exposed to increased levels of house dust mite allergen load at baseline (>10 μg/g dust). The results of this present study highlight the benefits of the sub-strategies of total bedroom control and nocturnal air purification of the breathing zone, with these sub-strategies suggesting improved outcomes, number of patients with improvements, and asthma symptom score. Chauhan et al^79^ reported the benefits of nocturnal temperature-controlled air purification on the exacerbation rate and quality of life in patients with severe asthma. The exacerbation rate was recently recommended by Kappen et al^80^ as the primary outcome for evaluating the effectiveness of allergen immunotherapy in allergic asthma, as well as the outcomes asthma symptom scores, and medication usage.

To the best of our knowledge, this is the first systematic review of house dust mite avoidance with a focus on the extraction of unbiased and precise summary statistics. The trials with a strong influence consistently showed environmentally divergent interventions among the other studies. The results of our study introduced new hypotheses in the debate on house dust mite avoidance. Our findings partly contrast with the conclusions by Gøtzsche and Johansen^4^ although the study was based on methods at a same level of evidence. Other recent reviews, such as those presented by Custovic et al,^81^ are based on lower levels of evidence. For instance, Custovic et al^81^ did not report any systematic process of selecting the included studies, nor did they include a quantitative synthesis of the results.

This study had some limitations. Due to the large number of subgroups, some of which were small in size or with a limited number of outcomes, the risk of false-positive findings by chance increased.^82^ This is particularly interesting as we observed notable benefits in the subgroups consists of a single study. In the sub-strategy of nocturnal air purification, our search did not include influential trials testing the effectiveness of a temperature-controlled laminar airflow during sleep for not focussing house dust mite-induced allergic asthma (for instance the study by Boyle et al^83^). Due to the small subgroups, we aimed to generate hypotheses instead of making claims regarding effect sizes. Our observations of effect sizes may have been affected by some clinical issues. As previously reported, the absence of significance in many effect sizes could possibly be related to the rather mild-to-moderate asthma status at baseline in many of the included patients, as well as the presence of co-sensitisation to cat and dog allergens.^6^ Another factor possibly affecting the effect sizes of concurrent bedroom interventions is the allergen exposure of patients outside the bedroom.^28^ Furthermore, only a few studies have reported on the GINA model for the management and classification of asthma.^7^ Finally, two of the observed benefits could not be judged because of their clinical relevance. The outcomes of both Zwemer et al^73^ and Carswell et al^47^ were based on obsolete asthma symptom scores, for which we do not know the MCID.

In our subgroup analysis based on environmental differences in avoidance strategies, including the (re)extraction of unbiased and precise outcomes, data from the sub-strategies of total bedroom control and nocturnal air purification of the breathing zone hypothetically provides benefits in regards to the number of asthmatic patients who experienced an improvement in their condition (patient-reported outcome), and the asthma symptom score. Paradoxically, these findings resulted from small-scaled single studies, as a result of our systematic collection of the data. Therefore, these hypotheses should be confirmed in future studies. Notably, the results of the large subgroup of the partial bedroom control sub-strategy confirm that it is no longer recommended in clinical practice. Therefore, future systematic reviews on the effectiveness of house dust mite avoidance should limit the study to focus on the sub-strategies of total bedroom control and nocturnal air purification of the breathing zone.

## Abbreviations

**Medical:** ACQ, asthma control questionnaire; AQLQ, the asthma quality of life questionnaire; ASS, asthma symptom score; FEV_1_, percentage of predicted forced expiratory volume in 1 s; FeNO, Fractionated exhaled NO levels; GINA, Global Initiative for Asthma; IgE, immunoglobulin E; MCID, minimal clinically important differences; PAQLQ, paediatric asthma quality of life questionnaire; PC_20_, histamine or methacholine concentration that caused a 20% decrease in the forced expiratory volume in 1 s; PRO, patient-reported outcome; RCT, randomised controlled trial; **Statistical:** 95% CI, 95% confidence interval; I^2^, heterogeneity statistic; MD, mean difference; n, sample size; NA, not applicable; RE, random-effects model; RR, risk ratio; rs, studentized residuals; SMD, standardised mean difference

## Acknowledgments

The authors are grateful to Mr. Rodney Q.J. van Boven for technical assistance with data extraction.

## Funding

No funding was received for this study.

## Availability of data and materials

The dataset generated during the current study is available from the corresponding author upon reasonable request.

## Authors’ contributions

Conceptualisation: FB, GJB, LA, MM, RGW, and NJ; formal analysis: FB and LA; investigation: FB, WB, and NJ; writing—original draft preparation: FB, GJB, LA, MM, WB, RGW, and NJ; writing—review and editing: FB, GJB, LA, MM, WB, RGW, and NJ. All authors have read and agreed to the published version of the manuscript.

## Ethics approval

This was a study of the literature. There was no testing of human subjects. The present meta-analysis is based on published data from clinical trials, all of them having their respective ethics evaluation and approvals.

## Author's consent for publication

All authors consented with the manuscript.

## Declaration of competing interest

FB received fees for lectures from St. Antonius Hospital, The Netherlands, as well as Asthma Association The Netherlands and Davos, and is a member of the Committee on Allergen Avoidance, V&VN Dutch Nurses' Association (unpaid). GJB reports consulting fees from 10.13039/100004325AstraZeneca, GSK, 10.13039/100004339Sanofi, 10.13039/100019719Chiesi, ALKAlbello, outside the submitted work. Furthermore, GJB is Chairman of the Asthma Section Dutch Lung Physicians, Secretary of the Working group Allergy ERS, and a member of the Scientific board Dutch Lung Foundation (all unpaid). The other authors declare no conflicts of interest in relation to this work.
