# Supplementary material for: House dust mite allergen avoidance strategies for the treatment of allergic asthma: A hypothesis-generating meta-analysis
Source: World Allergy Organ J. 2024 Jun 11;17(6):100919. doi: 10.1016/j.waojou.2024.100919 (PMC11223119; doi:10.1016/j.waojou.2024.100919)
Supplement: Multimedia component 1 [file mmc1.pdf]

Online Supplement A

Table S1. List of excluded studies that were included in the meta-analysis by Gøtzsche and Johansen (2008)

	**Author**	**Year**	**Our rationale**
1	Burr	1976	No useful data
2	Charpin	1990	Not in the English language
3	Chen	1996	Not in the English language
4	Cinti	1996	Not in the English language
5	Dietemann	1993	No useful data
6	Fang	2001	Not in the English language
7	Frederick	1997	No useful data
8	Geller-Bernst	1995	Not in the English language
9	Ghazala	2004	Not in the English language
10	Gillies	1987	No useful data
11	Howarth	1992	Only abstract
12	Jooma	1995	No useful data
13	Lee	2003	No useful data
14	Maesen	1997	Not in the English language
15	Manjra	1994	No useful data
16	Marks	1995	No useful data
17	Matthys	1996	No useful data
18	Mitchell	1980	No useful data
19	Popplewell	2000	No useful data
20	Sooltangos	1992	Only abstract
21	Van den Bemt	2004	No useful data
22	Van der Heide	1997B	Placebo group missing
23	Van der Heide	1999	No useful data
24	Verrall	1988	No useful data

25	Warner	2000	No useful data

References

1. Burr ML, St Leger AS, Neale E. Anti-mite measurements in mitesensitive adult asthma. A controlled trial. Lancet 1976;1:333-5.
2. Charpin D, Birnbaum J, Haddi E, N'Guyen A, Fondarai J, Vervloet D. Assessment of the eJectiveness of an acaricide, Acardust, in the treatment of acarian allergy Revue Française d'Allergologie 1990;30:149-55.
3. Chen CC, Hsieh K-H. EJects of Microstop-treated antimite bedding on children with mite-sensitive asthma. Acta Paediatrica Sinica 1996;37:420-7.
4. Cinti C, Canessa PA, Lavecchia MA, Capecchi V. Efficacia di un coprimaterasso e copricuscino 'antiacaro' nel controllo dell'asma dei pazienti allergici al dermatophagoides. Lotta Contro La Tuberculosi e Le Malattie Polmonari Sociali 1996;66:131-8.
5. Dietemann A, Bessot JC, Hoyet C, Ott M, Verot A, Pauli G. A double-blind, placebo controlled trial of solidified benzyl benzoate applied in dwellings of asthmatic patients sensitive to mites: clinical efficacy and effect on mite allergens. Journal of Allergy and Clinical Immunology 1993;91:738-46.
6. Fang Z, Cai Y, Wang L. [The efficacy of controlling of house dusts in attacks of mite sensitive asthmatics]. Zhonghua Jie He He Hu Xi Za Zhi 2001;24(11):685-9.
7. Frederick JM, Warner JO, Jessop WJ, Enander I, Warner JA. Effect of a bed covering system in children with asthma and house dust mite hypersensitivity. European Respiratory Journal 1997;10(2):361-6.
8. Geller-Bernstein C, Pibourdin JM, Dornelas A, Fondarai J. Efficacy of the acaricide: Acardust for the prevention of asthma and rhinitis due to dust mite allergy, in children. Allergie et Immunologie 1995;27:147-54.
9. Ghazala L, Schmid F , Helbling A , Pichler WJ, Pichler C E. Efficacy of house dust mite- and allergen-impermeable encasings in patients with house dust mite allergy. Allergologie 2004;27(1):26-34.
10. Gillies DRN, Littlewood JM, Sarsfield JK. Controlled trial of house dust mite avoidance in children with mild to moderate asthma. Clinical Allergy & Immunology 1987;17:105-11.
11. Howarth P, Lunn A, Tomkin S. Bedding barrier intervention in house dust mite respiratory allergy. Clinical & Experimental Allergy 1992;22:140.
12. Jooma OF, Weinberg EG, Berman D, Manjra AI, Potter PC. Accumulation of house-dust mite (Der-p-1) levels on mattress covers. South African Medical Journal 1995;85(10):1002-5.
13. Lee IS. Effect of bedding control on amount of house dust mite allergens, asthma symptoms, and peak expiratory flow rate. Yonsei Medical Journal 2003;44(2):313-22.
14. Maesen FPV, Sluysmans FG, Brombacher PJ, Smeets JJ. Ervaringen met het gebruik van luchtfiltratieapparatuur in de woonruimten van voor huisstof overgevoelige atopische patienten. Acta Tuberculosea et Pneumologica Belgica 1977;68:133-47.
15. Manjra A, Berman D, Toerien A, Weinberg EG, Potter PC. The effects of a single treatment of an acaricide, Acarosan, and a detergent, Metsan, on Der p 1 allergen levels in the carpets and mattresses of asthmatic children. South African Medical Journal 1994;84:278-80.
16. Marks GB, Tovey ER, Green W, Shearer M, Salome CM, Woolcock AJ. The effect of changes in house dust mite allergen exposure on the severity of asthma. Clinical & Experimental Allergy 1995;25:114-8.
17. Matthys H, Hupert A, Busch B. Dry air in bedrooms of patients with house dust mite-induced asthma. European Respiratory Journal 1996;9(Suppl 23):350s, abstract P2175.
18. Mitchell EA, Elliott RB. Controlled trial of an electrostatic precipitator in childhood asthma. Lancet 1980;2:559-61.
19. Popplewell EJ, Innes VA, Lloyd-Hughes S, Jenkins EL, Khdir K, Bryant TN, et al. The effect of high-efficiency and standard vacuum-cleaners on mite, cat and dog allergen levels and clinical progress. Pediatric Allergy & Immunology 2000;11(3):142-8.
20. Sooltangos S, Khodaboccus F, Baligadoo S, Leynadier F, Fadel R. Effect of house dust mites (HDM) avoidance measures on symptoms of asthmatic patients in Island of Mauritius. Journal of Allergy & Clinical Immunology 1992;89:259.
21. Van den Bemt L, Van Knapen L, De Vries MP, Jansen M, Cloosterman S, Van Schayck CP. Clinical effectiveness of a mite allergen-impermeable bed-covering system in asthmatic mitesensitive patients. Journal of Allergy & Clinical Immunology 2004;114(4):858-62.
22. van der Heide S, Kauffman HF, Dubois AE, de Monchy JG. Allergen reduction measures in houses of allergic asthmatic patients: eJects of air-cleaners and allergen-impermeable mattress covers. European Respiratory Journal 1997;10(6):1217-23.
23. van der Heide S, van Aalderen WM, Kauffman HF, Dubois AE, de Monchy JG. Clinical effects of air cleaners in homes of asthmatic children sensitized to pet allergens. Journal of Allergy & Clinical

Immunology 1999;104(2 Pt 1):447-51.
24. Verrall B, Muir DC, Wilson WM, Milner R, Johnston M, Dolovitch J. Laminar flow air cleaner bed attachment: a controlled trial. Annals of Allergy 1988;61:117-22.
25. Warner JA, Frederick JM, Bryant TN, Weich C, Raw GJ, Hunter C, et al. Mechanical ventilation and high-efficiency vacuum cleaning: a combined strategy of mite and mite allergen reduction in the control of mite-sensitive asthma. Journal of Allergy & Clinical Immunology 2000;105(1 Pt 1):75-82.

Table S2. List of included and excluded studies in the updated search.

	Author	Year	Included?	Rationale
1	Eick	2011	No	Not patients with house dust mite-allergic asthma
2	Glasgow	2011	No	Excluded before by Gøtzsche and Johansen
3	Maas	2011	No	Not tertiary prevention
4	Neymayr	2011	No	Not a clinical trial
5	Takaro	2011	No	Not randomized
6	Breysse	2012	No	Not a clinical trial
7	Celano	2012	No	Not patients with house dust mite-allergic asthma
8	El-Ghitany	2012	Yes	
9	Gehring	2012	No	Not tertiary prevention
10	Ho	2012	No	Only abstract
11	Masna	2012	No	Only abstract
12	Scott	2012	No	Not tertiary prevention
13	NCT	2013	No	Protocol issue
14	NCT	2013	No	Rhinitis
15	Tsurikisawa	2013	No	Not blinded
16	Hogaard	2014	No	Only abstract
17	NCT	2014	No	Duplicate
18	Hogaard	2014	No	Duplicate
19	Murray	2015	No	Duplicate
20	Smith	2015	No	Not blinded
21	Sumner	2015	No	Duplicate
22	Dimango	2016	No	Not patients with house dust mite-allergic asthma
23	NCT	2016	No	Protocol issue
24	Tsurikisawa	2016	No	Not blinded
25	Winn	2016	No	Not a clinical trial
26	Luo	2017	No	Only abstract
27	Murray	2017	Yes	
28	NCT	2017	No	Protocol issue
29	Morten	2018	No	Not patients with house dust mite-allergic asthma
30	Bjermer	2019	No	Not a clinical trial
31	Chen	2020	No	Protocol issue

32	Chen	2021	Yes	
33	Fong	2021	No	Protocol issue
34	Jia-Ying	2021	Yes	
35	Grant	2022	No	Multifaceted intervention

References

1. Eick SA, Richardson G. Investigation of different approaches to reduce allergens in asthmatic children's homes—The Breath of Fresh Air Project, Cornwall, United Kingdom. Sci Total Environ. 2011;409(19):3628–33.
2. Glasgow NJ, Ponsonby AL, Kemp A, Tovey E, Van Asperen P, McKay K, et al. Feather bedding and childhood asthma associated with house dust mite sensitisation: a randomised controlled trial. Arch Dis Child. 2011;96(6):541–7.
3. Maas T, Dompeling E, Muris JWM, Wesseling G, Knottnerus JA, van Schayck OCP. Prevention of asthma in genetically susceptible children: a multifaceted intervention trial focussed on feasibility in general practice. Pediatr Allergy Immunol. 2011;22(8):794–802.
4. Neumayr A, Niebauer E, Weber N, Haussinger K. Reduction of house dust mite allergens by using a silver-doped sleeping system. Allergologie. 2011;34(5):248–57.
5. Takaro TK, Krieger J, Song L, Sharify D, Beaudet N. The Breathe-Easy Home: the impact of asthma-friendly home construction on clinical outcomes and trigger exposure. Am J Public Health. 2011;101(1):55–62.
6. Breysse J, Wendt J, Dixon S, Murphy A, Wilson J, Meurer J, et al. Nurse case management and housing interventions reduce allergen exposures: the Milwaukee randomized controlled trial. Public Health Rep. 2011;126(SUPPL. 1):89–99
7. Celano MP, Holsey CN, Kobrynski LJ. Home-based family intervention for low-income children with asthma: a randomized controlled pilot study. J Fam Psychol. 2012;26(2):171–8.
8. El-Ghitany EM, El-Salam MMA. Environmental intervention for house dust mite control in childhood bronchial asthma. Environ Health Prev Med. 2012;17(5):377–84.
9. Gehring U, De Jongste JC, Kerkhof M, Oldewening M, Postma D, Van Strien RT, et al. The 8-year follow-up of the PIAMA intervention study assessing the effect of mite-impermeable mattress covers. Allergy. 2012;67(2):248–56.
10. Ho A, Vosicka K, Gore RB, Svensson P, Warner JO, Boyle RJ. Effect of temperature-controlled laminar airflow on symptoms and sleep quality in perennial allergic rhinitis. Clin Exp Allergy. 2012;42(12):1839–40.
11. Masna IAK, Yunus F, Sutoyo DK, The effect of air filter with balanced anion-cation usage on airway inflammation, asthma control, and lung function test of allergic asthma patients. Respirology. 2012; 17(6):6.
12. Scott M, Roberts G, Kurukulaaratchy RJ, Matthews S, Nove A, Arshad SH. Multifaceted allergen avoidance during infancy reduces asthma during childhood with the effect persisting until age 18 years. Thorax. 2012;67(12):1046–51.
13. Yunus F, Sutoyo DK. The effect of air filter with balanced anion-cation usage on airway inflammation, asthma control, and lung function test of allergic asthma patients. Respirology. 2012;17:6.
14. NCT. Cross-over study of the impact of Purotex covers on the concentration of house dust mite allergen in bedding and the quality of life in patients with allergic rhinitis to house dust mite. Clinicaltrialsgov[http://www.clinicaltrials.gov]. 2013.
15. Tsurikisawa N, Saito A, Oshikata C, Nakazawa T, Yasueda H, Akiyama K. Encasing bedding in covers made of microfine fibers reduces exposure to house mite allergens and improves disease management in adult atopic asthmatics. Allergy Asthma Clin Immunol. 2013;9(1):44.
16. Hogaard NV. P79-AsthmaVent-effect of mechanical ventilation on asthmacontrol in house dust mite allergic children with asthma. Clin Transl Allergy. 2014;4:132.
17. NCT. AsthmaVent—effect of mechanical ventilation on asthma control in children. https ://www.clinicaltrialsgov/show/nct02 06857 3. 2014.
18. Hogaard NV. AsthmaVent—effect of mechanical ventilation on asthmacontrol in house dust mite allergic children with asthma. Clin Transl Allergy. 2014;4(Suppl 1):42[P134].
19. Murray CS, Sumner H, Mycock M, Duxbury A, Custovic A, Simpson A. Preventing asthma exacerbations by allergen-impermeable bed covers in children: double-blind randomised placebo controlled trial. Allergy. 2015;70:75.

20. Smith H, Horney D, Goubet S, Jones C, Raza A, White P, et al. Pragmatic randomized controlled trial of a structured allergy intervention for adults with asthma and rhinitis in general practice. Allergy. 2015;70(2):203–11.
21. Sumner H, Begum H, Simpson A, Custovic A, Murray CS. The practicalities of using allergen impermeable bed covers in children with mite allergic asthma. Thorax. 2015;70:A122.
22. DiMango E, Serebrisky D, Narula S, Shim C, Keating C, Sheares B, et al. Individualized household allergen intervention lowers allergen level but not asthma medication use: a randomized controlled trial. J Allergy Clin Immunol Pract. 2016;4(4):671.e4–679.e4.
23. NCT. Impact of reduction of dust mite allergenic load on step down of inhaled corticosteroids in stable asthma. https ://www.clinicaltrialsgov/show/nct02 77362 8. 2016.
24. Tsurikisawa N, Saito A, Oshikata C, Yasueda H, Akiyama K. Effective allergen avoidance for reducing exposure to house dust mite allergens and improving disease management in adult atopic asthmatics. J Asthma. 2016;53(8):843–53.
25. Winn AK, Salo PM, Klein C, Sever ML, Harris SF, Johndrow D, et al. Efficacy of an in-home test kit in reducing dust mite allergen levels: results of a randomized controlled pilot study. J Asthma. 2016;53(2):133–8.
26. Luo J, Chen Z, Sun B. Efficacy of air purifier therapy in allergic asthma. Respirology. 2017;22(Supplement 3):97.
27. Murray CS, Foden P, Sumner H, Shepley E, Custovic A, Simpson A. Preventing severe asthma exacerbations in children a randomized trial of mite-impermeable bedcovers. Am J Respir Crit Care Med. 2017;196(2):150–8.
28. Jprn U. Study on indoor cleaning effect by air purifier and the influence on asthma. 2017.
29. Morten M, Collison A, Murphy VE, Barker D, Oldmeadow C, Attia J, et al. Managing asthma in pregnancy (MAP) trial: FENO levels and childhood asthma. J Allergy Clin Immunol. 2018;142(6):1765.e4–1772.e4.
30. Bjermer L, Eriksson G, Radner F, Peterson S, Warner JO. Time to onset of improvements in quality of life from temperature-controlled laminar airflow (TLA) in severe allergic asthma. Respir Med. 2019;147:19–25.
31. Chen M, Wu Y, Yuan S, et al. Research on allergic rhinitis improvement in asthmatic children after dust mite exposure reduction: a randomized, double-blind, cross-placebo study protocol. Trials. 2020;21(1).
32. Chen M, Wu Y, Yuan S, et al. Allergic Rhinitis Improvement in Asthmatic Children After Using Acaricidal Bait: A Randomized, Double-Blind, Cross-Placebo Study. Front Pediatr. 2021;9.
33. Nct. The Effect of Dyson Air Purifier in Improving Asthma Control. https://clinicaltrialsgov/show/NCT04729530. 2021.
34. Jia-Ying L, Li-Li O, Jing M, et al. Efficacy of air purifier therapy for patients with allergic asthma. Allergol Immunopathol (Madr). 2021;49(5):16-24.
35. Grant TL, McCormack MC, Peng RD, et al. Comprehensive home environmental intervention did not reduce allergen concentrations or controller medication requirements among children in Baltimore. J Asthma. 2022.

Table S3. Selection of environmentally relevant interventions in multiple arm trials (≥ 2 intervention arms)

**Author**	**Year**		**No of intervention arms**	**Description of the intervention**	**Sub strategy assessed?**	**Choice of the arm (yes/no) or intervention group?[1]**
Bahir	1997		2	A. Written instructions regarding environmental control (thoroughly house cleaning, hot washing of beddings)<br>B. Written instructions with active acaricide, applied twice to the mattress and the floors	Other strategy<br><br>Other strategy	No<br><br>Yes
Ehnert	1992		2	A. Matresses and carpets were treated with Acarosan on day 0, and at months 4 and 8.<br>B. mite impermeable covers fitted to the mattress, duvet, and pillow; carpets were sprayed with 3% tannic acid on day 0, and at months 4 and 8	Other strategy<br><br>Partial bedroom control	No<br><br>Yes
El-Ghitany	2012		3	A. Completely encasing mattresses and pillows; Washing the bedding weekly with hot water; Vacuuming the living room and	Other strategy[2]	No

				bedroom at least twice a week; Washing or refrigerating soft and furry toys once a week or excluding them from bedrooms; Removing carpets or vacuuming them more than once weekly; No pets.<br>B. Tannic acid 3% was provided for spraying the carpets and beddings twice weekly.<br>C. Combination of A and B.	Other strategy<br><br>Other strategy	No<br><br>Yes
Htut	2001		2	A. Heat-steam-heat treatment<br>B. Heat-steam-heat treatment combined with a special ventilation system installed in the loft.	Other strategy<br>Other strategy	No<br>Yes
Thiam	1999		2	A. mite impermeable covers fitted to the mattress, bolster (if any), and pillow; all carpets, rugs and furry toys were removed from the bedroom.<br>B. HEPA-filters were installed in the bedrooms.	Other strategy[2]<br><br>Air purification	Yes<br><br>No
Van der Heide	1997		2	A. Textile floors were treated with Acarosan powder. Mattresses	Other strategy	No

				were treated with Acarosan foam.<br>B. In the group of patients refusing chemical intervention, mattresses and pillows were encased with Intervent encasings.	Other strategy	Yes

1. The first step in the selection of the arm was to cover the mattress, duvette, and pillow (if described). The second step was to select the most comprehensive set of interventions from the remaining arms.
2. However, the duvets were not covered.

Table S4. Risk of bias judgements across the 35 randomised controlled trials included in the meta-analysis

**Author; year**	**Rationale if assessed a high risk of bias**
Antonicelli; 1991[1]	The control group received no blinded placebo intervention.
Bahir; 1997[2]	Sixteen children out of 46 dropped out (35%; judged high).
Burr; 1980A[3]	The control group received no blinded placebo intervention.
Burr; 1980B[4]	The control group received no blinded placebo intervention.
Carswell; 1996[5]	Forty-nine children out of 70 completed the study (30% drop out; judged high).
Chang; 1999[6]	The control group received no blinded placebo intervention.
Cloosterman; 1999[7]	Thirthy-two patients out of 157 dropped out (20%; judged high).
De Vries; 2007[8]	Allocation by grouping to the number on the list, in sequence of inclusion.
Dharmage; 2006[9]	The patients were randomized by the toss of a coin.
Dorward; 1988[10]	The control group received no blinded placebo intervention.
Ehnert; 1992[11]	
Halken; 2003[12]	Five children out of 52 dropped out (10%; judged high).
Htut; 2001[13]	Seven patients out of 30 dropped out (23%; judged high).
Huss; 1992[14]	
Korsgaard; 1983[15]	
Kroidl; 1998[16]	Seventy-eight patients out of 118 completed the study (34% drop out; judged high).
Luczynska; 2003[17]	Thirty-one patients out of 45 completed the study (31% drop out; judged high).
Marks; 1994[18]	Single blinding; only the treated subjects were unaware of the allocation.<br>35 subjects at baseline; follow-up: 5 NA's at 6 months (17%; judged high).
Reiser; 1990[19]	
Rijssenbeek; 2002[20]	Thirty patients out of 38 completed the study (21% drop out; judged high).
Sette; 1994[21]	
Shapiro;	Thirty-six patients out of 44 completed the study (18% drop out; judged high).

1999[22]	
Sheikh, 2002[23]	
Thiam; 1999[24]	The control group received no blinded placebo intervention.
Van der Heide, 1997[25]	
Walshaw; 1986[26]	Thirty patients out of 38 completed the study (21% drop out; judged high).
Warburton; 1994[27]	Twelve patients out of 13 completed the study (8% drop out in small sample; judged high).
Warner; 1993[28]	Fourteen patients out of 20 completed the study (30% drop out; judged high).
Woodcock; 2003[29]	
Wright; 2009[30]	Nineteen patients out of 101 dropped out (19%; judged high).
Zwemer; 1973[31]	Twelve patients out of 18 completed the study (33% drop out; judged high).
El-Ghitany; 2012[32]	
Murray; 2017[33]	Twelve months follow-up was completed in 85% (percentage incomplete 15%; risk judged high), no information on NA's.<br>Discrepancy between presented secondary outcomes in the publication and the protocol.
Chen; 2021[34]	
Jia ying; 2021[35]	The control group received no blinded placebo intervention.

FEV_1, forced expiratory volume in 1 s; NA, not applicable.

1. Antonicelli L, Bilo MB, Pucci S, Schou C, Bonifazi F. Efficacy of an air-cleaning device equipped with a high efficiency particulate air filter in house dust mite respiratory allergy. Allergy 1991; 46:594-600.
2. Bahir A, Goldberg A, Mekori YA, Confino-Cohen R, Morag H, Rosen Y, et al. Continuous avoidance measures with or without acaricide in dust mite-allergic asthmatic children. Ann Allergy Asthma Immunol 1997; 78:506-12.
3. Burr ML, Dean BV, Merrett TG, Neale E, St Leger AS, Verrier-Jones ER. Effects of anti-mite measures on children with mite-sensitive asthma: a controlled trial. Thorax 1980; 35:506-12.
4. Burr ML, Neale E, Dean BV, Verrier-Jones ER. Effect of a change to mite-free

bedding on children with mite-sensitive asthma: a controlled trial. Thorax 1980; 35:513-4.
5. Carswell F, Birmingham K, Oliver J, Crewes A, Weeks J. The respiratory effects of reduction of mite allergen in the bedrooms of asthmatic children--a double-blind controlled trial. Clin Exp Allergy 1996; 26:386-96.
6. Chang JH, Becker A, Ferguson A, Manfreda J, Simons E, Chan H, et al. Effect of application of benzyl benzoate on house dust mite allergen levels. Ann Allergy Asthma Immunol 1996; 77:187-90.
7. Cloosterman SG, Schermer TR, Bijl-Hofland ID, Van Der Heide S, Brunekreef B, Van Den Elshout FJ, et al. Effects of house dust mite avoidance measures on Der p 1 concentrations and clinical condition of mild adult house dust mite-allergic asthmatic patients, using no inhaled steroids. Clin Exp Allergy 1999; 29:1336-46.
8. de Vries MP, van den Bemt L, Aretz K, Thoonen BP, Muris JW, Kester AD, et al. House dust mite allergen avoidance and self-management in allergic patients with asthma: randomised controlled trial. Br J Gen Pract 2007; 57:184-90.
9. Dharmage S, Walters EH, Thien F, Bailey M, Raven J, Wharton C, et al. Encasement of bedding does not improve asthma in atopic adult asthmatics. Int Arch Allergy Immunol 2006; 139:132-8.
10. Dorward AJ, Colloff MJ, MacKay NS, McSharry C, Thomson NC. Effect of house dust mite avoidance measures on adult atopic asthma. Thorax 1988; 43:98-102.
11. Ehnert B, Lau-Schadendorf S, Weber A, Buettner P, Schou C, Wahn U. Reducing domestic exposure to dust mite allergen reduces bronchial hyperreactivity in sensitive children with asthma. J Allergy Clin Immunol 1992; 90:135-8.
12. Halken S, Host A, Niklassen U, Hansen LG, Nielsen F, Pedersen S, et al. Effect of mattress and pillow encasings on children with asthma and house dust mite allergy. J Allergy Clin Immunol 2003; 111:169-76.
13. Htut T, Higenbottam TW, Gill GW, Darwin R, Anderson PB, Syed N. Eradication of house dust mite from homes of atopic asthmatic subjects: a double-blind trial. J Allergy Clin Immunol 2001; 107:55-60.
14. Huss K, Squire EN, Jr., Carpenter GB, Smith LJ, Huss RW, Salata K, et al. Effective education of adults with asthma who are allergic to dust mites. J Allergy Clin Immunol 1992; 89:836-43.
15. Korsgaard J. Preventive measures in mite asthma. A controlled trial. Allergy 1983; 38:93-102.
16. Kroidl RF, Gobel D, Balzer D, Trendelenburg F, Schwichtenberg U. Clinical effects of benzyl benzoate in the prevention of house-dust-mite allergy. Results of a prospective, double-blind, multicenter study. Allergy 1998; 53:435-40.
17. Luczynska C, Tredwell E, Smeeton N, Burney P. A randomized controlled trial of mite allergen-impermeable bed covers in adult mite-sensitized asthmatics. Clin Exp Allergy 2003; 33:1648-53.
18. Marks GB, Tovey ER, Green W, Shearer M, Salome CM, Woolcock AJ. House dust mite allergen avoidance: a randomized controlled trial of surface chemical treatment and encasement of bedding. Clin Exp Allergy 1994; 24:1078-83.
19. Reiser J, Ingram D, Mitchell EB, Warner JO. House dust mite allergen levels and an anti-mite mattress spray (natamycin) in the treatment of childhood asthma. Clin Exp Allergy 1990; 20:561-7.
20. Rijssenbeek-Nouwens LH, Oosting AJ, de Bruin-Weller MS, Bregman I, de Monchy JG, Postma DS. Clinical evaluation of the effect of anti-allergic mattress covers in patients with moderate to severe asthma and house dust mite allergy: a randomised double blind placebo controlled study. Thorax 2002; 57:784-90.

21. Sette L, Comis A, Marcucci F, Sensi L, Piacentini GL, Boner AL. Benzyl-benzoate foam: effects on mite allergens in mattress, serum and nasal secretory IgE to Dermatophagoides pteronyssinus, and bronchial hyperreactivity in children with allergic asthma. Pediatr Pulmonol 1994; 18:218-27.
22. Shapiro GG, Wighton TG, Chinn T, Zuckrman J, Eliassen AH, Picciano JF, et al. House dust mite avoidance for children with asthma in homes of low-income families. J Allergy Clin Immunol 1999; 103:1069-74.
23. Sheikh A, Hurwitz B, Sibbald B, Barnes G, Howe M, Durham S. House dust mite barrier bedding for childhood asthma: randomised placebo controlled trial in primary care [ISRCTN63308372]. BMC Fam Pract 2002; 3:12.
24. Thiam DG, Tim CF, Hoon LS, Lei Z, Bee-Wah L. An evaluation of mattress encasings and high efficiency particulate filters on asthma control in the tropics. Asian Pac J Allergy Immunol 1999; 17:169-74.
25. van der Heide S, Kauffman HF, Dubois AE, de Monchy JG. Allergen-avoidance measures in homes of house-dust-mite-allergic asthmatic patients: effects of acaricides and mattress encasings. Allergy 1997; 52:921-7.
26. Walshaw MJ, Evans CC. Allergen avoidance in house dust mite sensitive adult asthma. Q J Med 1986; 58:199-215.
27. Warburton CJ, Niven RM, Pickering CA, Fletcher AM, Hepworth J, Francis HC. Domiciliary air filtration units, symptoms and lung function in atopic asthmatics. Respir Med 1994; 88:771-6.
28. Warner JA, Marchant JL, Warner JO. Double blind trial of ionisers in children with asthma sensitive to the house dust mite. Thorax 1993; 48:330-3.
29. Woodcock A, Forster L, Matthews E, Martin J, Letley L, Vickers M, et al. Control of exposure to mite allergen and allergen-impermeable bed covers for adults with asthma. N Engl J Med 2003; 349:225-36.
30. Wright GR, Howieson S, McSharry C, McMahon AD, Chaudhuri R, Thompson J, et al. Effect of improved home ventilation on asthma control and house dust mite allergen levels. Allergy 2009; 64:1671-80.
31. Zwemer RJ, Karibo J. Use of laminar control device as adjunct to standard environmental control measures in symptomatic asthmatic children. Ann Allergy 1973; 31:284-90.
32. El-Ghitany EM, Abd El-Salam MM. Environmental intervention for house dust mite control in childhood bronchial asthma. Environ Health Prev Med 2012; 17:377-84.
33. Murray CS, Foden P, Sumner H, Shepley E, Custovic A, Simpson A. Preventing Severe Asthma Exacerbations in Children. A Randomized Trial of Mite-Impermeable Bedcovers. Am J Respir Crit Care Med 2017; 196:150-8.
34. Chen M, Wu Y, Yuan S, Tang M, Zhang L, Chen J, et al. Allergic Rhinitis Improvement in Asthmatic Children After Using Acaricidal Bait: A Randomized, Double-Blind, Cross-Placebo Study. Front Pediatr 2021; 9:709139.
35. Jia-Ying L, Li-Li O, Jing M, Xin-Yuan L, Li-Min F, Hai-Cheng L, et al. Efficacy of air purifier therapy for patients with allergic asthma. Allergol Immunopathol (Madr) 2021; 49:16-24.
